# Effects of control measures on the spread of LA-MRSA among Danish pig herds between 2006 and 2015 – a simulation study

**DOI:** 10.1038/s41598-018-37075-8

**Published:** 2019-01-24

**Authors:** Jana Schulz, Anette Boklund, Nils Toft, Tariq Halasa

**Affiliations:** 1 Technical University of Denmark, National Veterinary Institute, Kemitorvet, Building 204, 2800 Kgs., Lyngby, Denmark; 2 University of Copenhagen, Faculty of Health Sciences, Grønnegårdsvej 8, 1870 Frederiksberg C, Denmark

## Abstract

There has been a rapid increase in Danish pig herds testing positive for livestock-associated Methicillin-resistant *Staphylococcus aureus* (LA-MRSA) since the first screening in 2008. Despite a national action plan to control LA-MRSA in the Danish pig population, 88% of pig herds tested positive in a 2016 cross-sectional study of 57 herds. The national action plan was initiated in April 2015 and aimed to reduce the spread of LA-MRSA among pig herds. However, its success is uncertain. We used a simulation model mimicking the spread of LA-MRSA among pig herds between 2006 and 2015 to evaluate the impact of control strategies if these had these been implemented in 2007 or 2010. The strategies were combinations of the following control measures: (1) a reduced number of herds using high-risk antibiotics, (2) a reduced probability of indirect transmission among herds via humans, (3) movement restrictions, and (4) voluntary eradication in 5–7.5% of the herds. Almost all tested control strategies simulated a reduction in the spread of LA-MRSA. The combination of two, three or four intervention strategies showed additive effects and led to larger reductions in the predicted herd prevalence. In addition, the prevalence of LA-MRSA-positive herds at the time when control measures were initiated influenced the effects of the control strategies. Combining the simulated control measures can be considered in future action plans to control LA-MRSA.

## Introduction

Methicillin-resistant *Staphylococcus (S.) aureus* (MRSA) is a group of *S. aureus* that is resistant to most β-lactam antibiotics^1^. The main reservoir for livestock-associated MRSA (LA-MRSA) is the pig population, though it has also been found in humans and other animal species^2^. In humans, (LA-) MRSA can cause severe infections in children, elderly or immunosuppressed people. The number of LA-MRSA cases in humans has increased in recent years^3^. Transmission from livestock to humans has been established^4,5^, so limiting the spread of LA-MRSA in the pig population may limit the number of LA-MRSA cases in humans.

The first detection of LA-MRSA in Danish pig farms was in isolates from samples taken in 2006^6^. In 2008, a survey conducted in 26 European countries by the European Food Safety Authority (EFSA) found 3% of Danish production herds, but no Danish breeding herds, were positive for LA-MRSA type CC398^7^. However, a 2014 national survey found a prevalence of 63% in breeding herds and 68% in slaughter pig herds^8^, and a similar survey from 2016 found a prevalence of 88% in finisher herds^3^.

Danish pig production has a pyramidal structure with breeding herds at the top, production herds in the middle and slaughterhouses at the bottom^9^. Pig movements mainly occur vertically (i.e. from the top to the bottom of the pyramid) in accordance with pig production, but horizontal connections (i.e. among herds of the same herd type) also exist^9^. Pig movements were identified as an important route for the spread of LA-MRSA among pig herds^10,11^. The Danish Veterinary and Food Administration (DVFA) published an action plan for controlling LA-MRSA in April 2015, based on recommendations from a risk assessment^12^. This action plan aimed to reduce the use of antibiotics in pig production by 15% from 2015 to 2018, thereby reducing levels of LA-MRSA in pig herds. Catry *et al*.^13^ described potential control measures to limit the spread of LA-MRSA among pig herds based on risk factors for LA-MRSA spread. They suggested: (1) improved hygiene within herds and during transport, beginning with the breeding herds and followed by the rest of the production chain, and (2) prevention of pig movements from MRSA-positive to MRSA-negative herds.

Schulz *et al*.^14^ developed an agent-based Monte Carlo simulation model of the spread of LA-MRSA among pig herds in order to study the epidemic behaviour and to identify the driving factors in LA-MRSA spread among pig herds. The model suggested that the spread of LA-MRSA could be explained by three transmission routes: animal movements, indirect contact and unexplained introductions. None of the three transmission routes on their own were able explain the rapid increase in LA-MRSA prevalence in Denmark. However, combining all three routes under the model assumptions mimicked a development of LA-MRSA-positive herds similar to the trend observed in Denmark. Both the frequency and effectiveness of indirect contact with humans visiting more than one herd on the same day were identified as sensitive parameters in the model presented by Schulz *et al*.^14^. The model can be used to assess which control measures could have been used to control the spread of LA-MRSA among herds. Retrospective studies on how an epidemic in a country/region could have had been influenced may aid in controlling future epidemics in the same or in similar areas.

The objective of this study was to investigate how the spread of LA-MRSA of type CC398 (hereinafter referred to as LA-MRSA) among Danish pig herds between 2006 and 2015 could have been influenced by: (1) a reduced number of herds using high-risk antibiotics, (2) a reduced probability of indirect transmission via humans visiting more than one herd per day (reflecting high levels of biosecurity), (3) movement restrictions between LA-MRSA-positive and negative herds, and (4) voluntary eradication of MRSA in 5–7.5% of the herds. Additionally, we compared two starting points for these control actions to evaluate the impact on the reduction of LA-MRSA spread.

## Materials and Methods

### Simulation model

**Figure 1:**
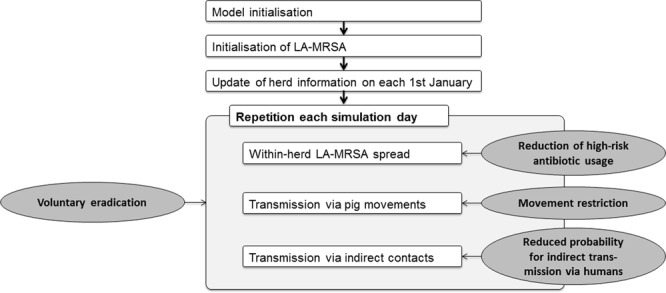
Structure of the LA-MRSA spread simulation model (adapted from Schulz *et al*.^14^). The original structure is enhanced by four potential control strategies (dark grey ellipses).

We used an agent-based Monte Carlo simulation model mimicking the spread of LA-MRSA among Danish pig herds between 2006 and 2015. Figure 1 illustrates the structure of the original model, which is described in detail by Schulz *et al*.^14^ Herd information on Danish pig herds and movement data from 1^st^ January 2006 to 31^st^ December 2015 were used as the basis for modelling.

Within-herd spread was simulated using a three-compartment SIS model with different transmission rates within the three compartments of sows, weaners and finishers, and with high- and low-risk transmission routes between these compartments. PERT distributions with higher minimum, mode and maximum values were used for herds using high-risk antibiotics (Table 1). A stochastic and time-discrete simulation process was implemented to mimic spontaneous recovery from LA-MRSA (i.e. colonised pigs could randomly be cleared of LA-MRSA at any time, according to pre-defined cure rates).

**Table 1:** Assumed values for a PERT distribution to define herd-specific transmission rates based on the use of high-risk antibiotics, adapted by Broens *et al*.^17^. The original table was presented in Schulz *et al*.^14^.

Use of high-risk antibiotics	Within-compartment transmission rate	Low-risk between-compartment transmission rate	High-risk between-compartment transmission rate
no	min = 0.111	min = 0.00175	min = 0.07184
	max = 0.856	max = 0.00301	max = 0.48155
	mode = 0.307	mode = 0.00233	mode = 0.18301
yes	min = 0.211	min = 0.00330	min = 0.13689
	max = 2.924	max = 0.01029	max = 1.64515
	mode = 0.784	mode = 0.00583	mode = 0.46796

Between-herd spread was modelled via two routes: direct and indirect contact among pig herds. Direct transmissions were modelled using data on animal movements registered in the Central Husbandry Register (CHR)^15^. Registered movements among herds were used directly in the model. In the original model, the number of registered sows, weaners and finishers and the number of positive pigs in each of these compartments was recorded for each herd during the simulation. Each pig movement record consisted of the date of the movement, the number of pigs moved (batch size) and the types of pigs moved out of the sending herd and into the receiving herd (i.e. sows, weaners or finishers could be moved out of the sending herd and sows, weaners or finishers could be moved into the receiving herd). Pig movements were modelled as follows:

1. If the sending herd was negative for LA-MRSA (i.e. the number of positive pigs was zero in each of the three compartments), no pigs were moved because transmission was not possible, and herd sizes were kept constant during each simulated year.
2. If the sending herd was LA-MRSA positive, the number of LA-MRSA-positive pigs in the movement batch was calculated based on the prevalence of the sending herd (i.e. if the sending herd had a within-herd prevalence of 50%, the prevalence in the movement batch was also assumed to be 50%). In the receiving herd, an increased prevalence was calculated based on the number of positive pigs in the receiving herd plus the number of positive pigs in the movement batch.

Indirect contact was mimicked as the transmission of LA-MRSA via humans visiting more than one pig herd per day and via trucks that collect pigs for slaughter from more than one herd on the same day. While data for the collection of pigs sent to the abattoir was available in the movement register and these were used to calculate herd-specific lambdas, herd-level data did not exist for visitors. This contact type was modelled as a Poisson distribution with the same mean (λ) for all indoor or outdoor herds (Table 2). The probability of infection via indirect contact with LA-MRSA-positive herds was modelled as a PERT distribution (Table 2).

**Table 2:** Overview of simulation parameters and default values used in the LA-MRSA spread model developed by Schulz *et al*.^14^. Only those parameters related to transmission via indirect contact that varied in the presented study are shown.

Variable name	Default value	Description	Reference
**Modelling disease spread among herds**			
*Spread via indirect contact*			
*λ*_*in*_	0.256	Average daily probability of indirect contact originating from an LA-MRSA-positive indoor herd	Adjusted based on Boklund *et al*.^27^
*λ*_*out*_	0.1864	Average daily probability of indirect contact originating from an LA-MRSA-positive outdoor herd	Adjusted based on Boklund *et al*.^27^
*prob*_*in*_	PERT (min = 0.001, max = 0.01, mode = 0.005071)	Probability of infection via contact from an LA-MRSA-positive indoor herd	Expert opinion
*prob*_*out*_	PERT (min = 0.001, max = 0.01, mode = 0.0035)	Probability of infection via contact from an LA-MRSA-positive outdoor herd	Expert opinion
*prob*_*a*_	PERT (min = 0.001, max = 0.01, mode = 0.004714)	Probability of infection via abattoir movements	Expert opinion

Herds at different addresses could be owned by the same farmer, and transmission among these pig herds was modelled to mimic the contact and potential transmission of LA-MRSA via shared workers or equipment. If a herd was positive for LA-MRSA, transmission was implemented among all herds owned by the same farmer. Therefore, the herd prevalence was calculated for the positive herd and a low-risk transmission rate was used for between-compartment transmission in order to model the spread between the positive herd to the sow (or weaner or finisher) section of the other herds owned by the same farmer (Table 1).

In the original study by Schulz *et al*.^14^, 17 initialisation scenarios were simulated in which LA-MRSA was introduced to pig herds in 2006 and, depending on the scenario, further introductions followed in subsequent years. The predicted herd prevalence was compared to LA-MRSA screening results in 2008 and 2014. We defined a new initialisation scenario for the introduction of LA-MRSA in the first years of the simulation period, aiming at a median herd prevalence between 60% and 70% in 2015. We selected 400 production herds and 10 breeding and multiplier herds at random to be LA-MRSA positive in 2006. A second introduction was modelled in 2009, again by random selection of 400 production herds and 10 breeding and multiplier herds positive for LA-MRSA.

In the following scenarios, the predicted herd prevalence on 31^st^ December 2012 was the basis for comparison among different control strategies.

### Control strategies

To simulate different interventions and to evaluate their performance, the initial model developed by Schulz *et al*.^14^ was enhanced. Four control measures were simulated, either separately or in different combinations. All control measures were initiated on 1^st^ January 2007 (initiation date) and continued until the end of the simulation. To investigate the effect of starting date, i.e. the effect of the initial prevalence of MRSA-positive herds, all scenarios were also run with 1^st^ January 2010 as the initiation date for the simulated control strategy. The results were measured as the prevalence of LA-MRSA-positive herds on 31^st^ December 2012 (for initialisation in 2007) and 31^st^ December 2015 (for initialisation in 2010). This enabled us to compare the effects after a similar time period. If several control measures were combined, all control measures were initiated on the same date. In addition, this allowed us to quantify the effects of control measures under a higher initial LA-MRSA prevalence compared to the initial prevalence in 2007.

The relative reduction was calculated as the proportion *R*_*s*_ for each scenario *s*, calculated as:

$${{R}}_{{s}}=\frac{\overline{{Pre}{{v}}_{{d}}-}\overline{{Pre}{{v}}_{{s}}}}{\overline{{Pre}{{v}}_{{d}}}},$$

with $\overline{{Pre}{{v}}_{{s}}}$ as the predicted median prevalence of scenario *s* and $\overline{{Pre}{{v}}_{{d}}}$ as the predicted median prevalence of the default scenario.

#### Reduction of high-risk antibiotic use (AB)

Tetracycline and β-lactam antimicrobials were defined as high-risk antibiotics, as these antimicrobial classes select for LA-MRSA CC398^16^ and were shown to affect its transmission^17^. The original model included information on the prescription of β-lactams or tetracycline at herd level^14^. Herds that received prescriptions for these antibiotics were modelled with higher within-herd transmission rates (value high-transmission) compared to herds that did not receive prescriptions (value low-transmission; Table 1). In the scenarios that used this control measure, 50% or 100% of herds that received prescriptions for high-risk antibiotics on the initiation date were randomly chosen. From the initiation date until the end of the simulation period, cessation of high-risk antimicrobial use was simulated by changing the within-herd dynamics of these herds using transmission rates for herds that did not use high-risk antibiotics.

#### Reduced probability for indirect transmission via humans (ProbIT)

In scenarios using this control measure, we reduced the minimum, maximum and mode value of the default PERT distributions by 50% or 75% in all herds (Table 2).

#### Movement restriction (MR)

To limit the spread of LA-MRSA via pig movements, a potential control option would be to prohibit movements from LA-MRSA-positive to LA-MRSA-negative herds. In this case, the status of the herds must be known. We enhanced the initial model by simulating periodic LA-MRSA screenings within the herds. Testing was simulated by nasal swabs with a sensitivity of 78% and a specificity of 99.9%^18^. We randomly assigned test results to the simulated herds based on assumed test characteristics of the true status of the herds on the day of an LA-MRSA screening.

For all movements, we then checked the following cases:

1. If the sending herd had a negative test result, pigs were moved according to the movement data.
2. If the sending and receiving herds were simulated to have positive test results, pigs were also moved according to the movement data.
3. If the sending herd had a positive test result, but the receiving herd had a negative test result, we assumed that the pigs were moved to another LA-MRSA-positive herd. For all LA-MRSA-positive herds, we checked for a potential new receiver of the same herd category (dependent on the number of registered sows, weaners and finishers) and randomly selected one, if available. If there were no potential new receivers, we assumed that the pigs were exported and therefore disregarded the movement.

We ran two scenarios to mimic movement restrictions: testing all herds once per year and testing all herds four times per year. We assumed that all herds were tested on the same day.

#### Voluntary eradication (Erad)

Mimicking an eradication process (i.e., depopulation followed by cleaning and disinfection) also required testing the herds, as only herds testing positive for LA-MRSA would initiate an eradication programme. We assumed that 7.5% of the breeding and multiplier herds and 5% of all other herd types would begin eradication after testing positive for LA-MRSA. These herds were chosen randomly out of all breeding and multiplier (other herds) that tested positive for LA-MRSA. The eradication process lasted between 168 and 378 simulation days, depending on the production type of the herd (Table 3). These values were based on experience of previous eradication programmes performed in Danish pig herds (personal communication, Finn Udesen – SEGES, Danish Agriculture & Food Council). During this time period, no movements (either in or out) were performed. We ignored these movements, assuming that the herd did not send pigs to other herds, except for slaughter or culling. Moreover, we assumed that the herd would have been restocked with LA-MRSA-negative pigs. As soon as the eradication period ended, registered in-coming and out-going movements were modelled as implemented in the original model. We ran this control measure assuming that herds would be screened for LA-MRSA once per year. When voluntary eradication was combined with movement restrictions based on testing all herds four times per year, herds were sampled four times per year as well as at the start of the eradication process. An upper limit of 25% of all registered breeding and multiplier herds was set. If this limit was exceeded, no additional breeding and multiplier herds initiated eradication. No limit was set for other herd types.

**Table 3:** Assumed duration of the eradication process, dependent on herd categories based on the registered number of sows and finishers.

Production type	Description	Assumed duration of eradication process
Sow herd	<5 finishers per sow	266 days
Integrated herd	5–7.7 finishers per sow	378 days
Finisher herd	>7.5 finishers per sow	168 days

All control strategies were added to the original model individually and combined in all possible combinations.

Simulation modelling and graphical presentation of results were performed in R version 3.2.2 - “Fire Safety“^19^. Like the original model, all simulations were run with 500 iterations to cover any extra variability in the different scenarios.

## Results

The default initialisation scenario without control measures led to a predicted median herd prevalence of 47% on 31^st^ December 2012 and 62% on 31^st^ December 2015 (Table 4). On 1^st^ January 2007, the median prevalence was 4% [90% prediction interval: 3–7%]. Control strategies with this initiation date are presented in Table 4. No reduction in median prevalence was observed when the number of herds using high-risk antibiotics was reduced by 50% (Table 4, Scenario 1.1: AB (50%)). However, a relative reduction of 38% was observed if all herds reduced the use of high-risk antibiotics (Table 4, Scenario 1.2: AB (100%)). Furthermore, these scenarios showed larger variation compared to the default scenario. Reducing the probability of effective indirect contact from LA-MRSA-positive herds by 50% or 75% (Scenarios 1.3 and 1.4: ProbIT (75%) and ProbIT (100%)) led to reductions in the median prevalence to 37% and 31%, respectively (Table 4). Movement restrictions did not lead to a reduction in the predicted median prevalence when it was based on testing herds once per year (Table 4, Scenario 1.5: MR (1/year)), while a relative reduction of 22% was observed when movement restrictions were based on four yearly screenings (Table 4, Scenario 1.6: MR (4/year)). Voluntary eradication (Scenario 1.7: Erad (1/year)) led to a limited reduction in the median prevalence to 43%.

**Table 4:** Predicted median prevalence of the default scenario and the four individual control measures 6 years after initiation of the control programme.

Scenario ID	Scenario acronym	Predicted median herd prevalence in % on 31^st^ December 2012 with initialisation of control on 1^st^ January 2007 [90% prediction interval] (relative reduction)	Predicted median herd prevalence in % on 31^st^ December 2015 with initialisation of control on 1^st^ January 2010 [90% prediction interval] (relative reduction)
**No control measures**			
0	Default	47 [42–52]	62 [59–65]
**Single control measures**			
1.1	AB (50%)	47 [30–61] (0%)	59 [46–70] (6%)
1.2	AB (100%)	29 [13–44] (38%)	48 [26–60] (24%)
1.3	ProbIT (50%)	37 [31–43] (21%)	57 [52–60] (9%)
1.4	ProbIT (75%)	31 [26–37] (33%)	53 [48–57] (15%)
1.5	MR (1/year)	47 [43–53] (0%)	63 [60–66] (−1%)
1.6	MR (4/year)	37 [32–42] (22%)	55 [51–58] (12%)
1.7	Erad (1/year)	43 [38–49] (8%)	59 [55–62] (6%)

The scenario acronyms are described in the main text.

In the next step, we combined two control scenarios. We observed an additive effect that was slightly larger when the combination included a reduction in high-risk antibiotic use in all herds (Table 5, Scenarios 2.6–2.10). The variance increased in all scenarios that included the reduction of high-risk antibiotics. The lowest median herd prevalence (15%) was found when reduced high-risk antibiotic use in 100% of the herds was combined with a 75% reduction in the probability of effective indirect contact from LA-MRSA-positive herds (Table 5, Scenario 2.7).

**Table 5:** Predicted median prevalence of the default scenario and a combination of two control measures 6 years after the initiation of the control programme.

Scenario ID	Scenario acronym		Predicted median herd prevalence in % on 31^st^ December 2012, initialisation of control on 1^st^ January 2007 [90% prediction interval] (relative reduction)	Predicted median herd prevalence in % on 31^st^ December 2015, initialisation of control on 1^st^ January 2010 [90% prediction interval] (relative reduction)
**Combination of two control measures**				
2.1	AB (50%)	ProbIT (50%)	36 [22–49] (24%)	52 [39–64] (16%)
2.2		ProbIT (75%)	30 [17–42] (37%)	49 [36–60] (22%)
2.3		MR (1/year)	47 [30–61] (0%)	59 [48–70] (5%)
2.4		MR (4/year)	35 [22–50] (25%)	51 [38–63] (17%)
2.5		Erad (1/year)	42 [26–56] (10%)	55 [41–67] (12%)
2.6	AB (100%)	ProbIT (50%)	22 [11–35] (54%)	41 [23–55] (34%)
2.7		ProbIT (75%)	15 [7–26] (68%)	38 [22–50] (40%)
2.8		MR (1/year)	30 [15–46] (36%)	48 [28–63] (22%)
2.9		MR (4/year)	21 [9–33] (56%)	38 [20–50] (39%)
2.10		Erad (1/year)	26 [11–40] (45%)	41 [24–57] (34%)
2.11	ProbIT (50%)	MR (1/year)	38 [32–43] (19%)	58 [53–61] (8%)
2.12		MR (4/year)	28 [23–33] (41%)	49 [44–53] (22%)
2.13		Erad (1/year)	33 [28–39] (29%)	52 [48–56] (16%)
2.14	ProbIT (75%)	MR (1/year)	32 [27–39] (31%)	54 [49–58] (14%)
2.15		MR (4/year)	23 [19–29] (50%)	45 [40–49] (28%)
2.16		Erad (1/year)	28 [23–34] (41%)	48 [42–52] (24%)
2.17	MR (1/year)	Erad (1/year)	29 [25–34] (37%)	46 [42–49] (26%)
2.18	MR (4/year)	Erad (4/year)	21 [18–25] (55%)	36 [32–40] (42%)

The scenario acronyms are described in the main text.

The combination of three control measures led to a maximum reduction in the simulated median prevalence to 10% herd prevalence when the reduction of high-risk antibiotics in all herds, movement restrictions based on four tests per year, and voluntary eradication scenarios were combined (Table 6, Scenario 3.16). In 14 of 20 scenarios, the relative reduction was higher than 50%.

**Table 6:** Predicted median prevalence of the default scenario and a combination of three control measures 6 years after the initiation of the control programme.

Scenario ID	Scenario acronym			Predicted median herd prevalence in % on 31^st^ December 2012 [90% prediction interval] (relative reduction)	Predicted median herd prevalence in % on 31^st^ December 2015 [90% prediction interval] (relative reduction)
**Combination of three control measures**					
3.1	AB (50%)	ProbIT (50%)	MR (1/year)	36 [22–49] (23%)	53 [40–65] (14%)
3.2			MR (4/year)	26 [16–38] (44%)	44 [32–56] (29%)
3.3			Erad (1/year)	32 [19–46] (31%)	48 [34–61] (23%)
3.4		ProbIT (75%)	MR (1/year)	32 [20–43] (33%)	50 [38–61] (20%)
3.5			MR (4/year)	21 [13–33] (55%)	40 [28–50] (36%)
3.6			Erad (1/year)	26 [15–38] (44%)	44 [31–55] (30%)
3.7		MR (1/year)	Erad (1/year)	29 [18–41] (39%)	42 [32–53] (32%)
3.8		MR (4/year)	Erad (4/year)	20 [11–31] (57%)	32 [21–42] (48%)
3.9	AB (100%)	ProbIT (50%)	MR (1/year)	22 [10–35] (53%)	42 [25–56] (32%)
3.10			MR (4/year)	15 [7–25] (68%)	33 [14–44] (48%)
3.11			Erad (1/year)	38 [32–43] (61%)	35 [20–49] (43%)
3.12		ProbIT (75%)	MR (1/year)	19 [7–30] (59%)	38 [22–49] (39%)
3.13			MR (4/year)	13 [6–22] (72%)	29 [16–39] (53%)
3.14			Erad (1/year)	15 [5–26] (68%)	31 [16–43] (50%)
3.15		MR (1/year)	Erad (1/year)	16 [8–28] (65%)	30 [15–41] (52%)
3.16		MR (4/year)	Erad (4/year)	10 [4–18] (79%)	21 [10–31] (67%)
3.17	ProbIT (50%)	MR (1/year)	Erad (1/year)	21 [17–25] (55%)	39 [34–42] (38%)
3.18		MR (4/year)	Erad (4/year)	15 [12–17] (69%)	29 [25–33] (53%)
3.19	ProbIT (75%)	MR (1/year)	Erad (1/year)	17 [14–21] (64%)	34 [29–39] (46%)
3.20		MR (4/year)	Erad (4/year)	12 [9–14] (75%)	25 [21–29] (60%)

The scenario acronyms are described in the main text.

Combining all four control measures showed the highest potential to reduce the simulated prevalence compared to the default scenario (Table 7). The smallest median prevalence was estimated at 6% (90% prediction interval: 2–10%) and was observed with the combination of antibiotic reduction in all herds, a 75% reduction in the probability of effective indirect transmission via humans, movement restrictions based on testing all herds four times per year and voluntary eradication (Table 7, Scenario 4.8). LA-MRSA was not cleared from all simulated herds following the set-up of control measures in any of the simulation scenarios.

**Table 7:** Predicted median prevalence of the default scenario and the combination of four control measures 6 years after the initiation of the control programme.

Scenario ID	Scenario acronym				Predicted median herd prevalence in % on 31^st^ December 2012 [90% prediction interval] (relative reduction)	Predicted median herd prevalence in % on 31^st^ December 2015 [90% prediction interval] (relative reduction)
**Combination of four control measures**						
4.1	AB (50%)	ProbIT (50%)	MR (1/year)	Erad (1/year)	20 [12–30] (56%)	34 [24–44] (45%)
4.2			MR (4/year)	Erad (4/year)	13 [8–22] (72%)	25 [16–34] (61%)
4.3	AB (50%)	ProbIT (75%)	MR (1/year)	Erad (1/year)	16 [9–24] (65%)	30 [21–38] (53%)
4.4			MR (4/year)	Erad (4/year)	11 [6–16] (77%)	21 [13–30] (66%)
4.5	AB (100%)	ProbIT (50%)	MR (1/year)	Erad (1/year)	11 [5–19] (77%)	23 [14–33] (62%)
4.6			MR (4/year)	Erad (4/year)	7 [3–13] (86%)	16 [6–24] (74%)
4.7	AB (100%)	ProbIT (75%)	MR (1/year)	Erad (1/year)	9 [5–15] (80%)	21 [11–29] (66%)
4.8			MR (4/year)	Erad (4/year)	6 [2–10] (86%)	13 [5–19] (79%)

The scenario acronyms are described in the main text.

Simulating control measure initiation on 1^st^ January 2010 led to comparable tendencies in the effects of single interventions and combinations of the tested control options. The median prevalence on the initiation date was 21% [90% prediction interval: 18–34%]. In all scenarios, the predicted herd prevalence was higher when control was initiated in 2010, compared to the scenarios with an initiation date of 1^st^ January 2007 (Tables 4–7). The smallest median herd prevalence 6 years after initiation in 2010 was 13% (90% prediction interval: 5–19%), compared to 6% (90% prediction interval: 2–10%) for controls initiated in 2007 in the scenario combining all four control measures (Scenario 4.8). Initiating control measures in 2007 generally led to higher relative reduction rates than the same control strategy started in 2010 (Tables 4–7). However, reduction rates were still fairly high when the combination of four control measures was initiated in 2010.

**Figure 2:**
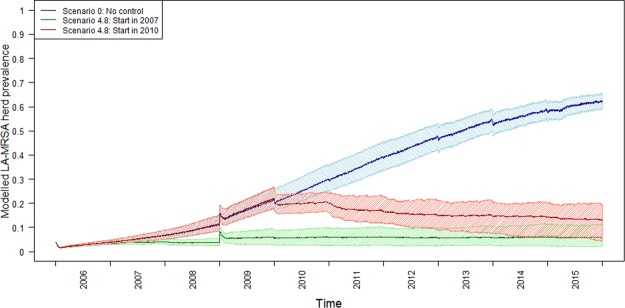
Predicted LA-MRSA herd prevalence over the whole study period from 1^st^ January 2006 to 31^st^ December 2015 for the following three scenarios: (1) Default (blue), (2) Scenario 4.8 (AB (100%) + ProbIT (75%) + MR (4/year) + Erad (4/year)) for control measures starting on 1^st^ January 2007 (green), and (3) Scenario 4.8 for control measures starting on 1^st^ January 2010 (red). Dark lines represent the predicted median herd prevalence, the light dashed areas represent the 90% prediction interval. The scenario acronyms are described in the main text.

Figure 2 shows the development of the predicted LA-MRSA herd prevalence for the default scenario (Scenario: 0), and for the scenario with the highest reduction in LA-MRSA herd prevalence (Scenario: 4.8) for both of the control strategy initiation dates.

## Discussion

LA-MRSA spread among Danish pig herds was modelled using four potential control options. The results showed that initiating intensive and combined control measures in 2007 would have led to a slower increase in the LA-MRSA herd prevalence (Tables 4–7). In particular, the combination of all four implemented control measures showed the potential to limit the spread among pig herds. Nevertheless, LA-MRSA was not cleared from all herds during the study period for any of the tested scenarios. Initiating control measures in 2010 also showed a reduction in the predicted herd prevalence of LA-MRSA. However, the relative reductions were smaller when compared to simulating the start of control in 2007 (Tables 4–7).

Reducing the use of high-risk antibiotics such as β-lactams and tetracycline has been shown to reduce transmission rates for the within-herd spread of LA-MRSA^17^. We found that reducing the proportion of herds using high-risk antibiotics had a limiting effect on the between-herd spread of LA-MRSA. This might be related to the lower within-herd prevalence and thus to a lower risk of LA-MRSA transmission among herds. Our results correspond to the findings of Sørensen *et al*.^20^, who used a mechanistic simulation model to show that reducing antimicrobial consumption reduced the prevalence of LA-MRSA within a farrow-to-finisher herd, but that bacteria was not eradicated. Reducing the within-herd prevalence would reduce the probability of infection following contact with a susceptible herd, leading to a reduction in the between-herd prevalence. However, as the transmission rate is not zero after a reduction in the use of high-risk antibiotics^15^, the pathogen can still spread and hence eradication did not occur.

All scenarios that included a reduction of high-risk antibiotics led to increased variance in the predicted prevalence on 31^st^ December 2015. Low transmission rates might have led to a higher number of herds in which LA-MRSA faded out after introduction. More precisely, LA-MRSA might have been cleared from pigs after the introduction of control measures and before transmission within or between the compartments occurred. This effect might therefore have reduced the spread of LA-MRSA among pig herds and resulted in lower predicted herd prevalences. On the other hand, if LA-MRSA was established in a pig herd, the within-herd prevalence reached a similar level to that of herds using high-risk antibiotics^14^, meaning that the spread from these herds to other herds was not affected. This might explain the larger variation in herd prevalence at the end of the study period compared to the default scenario.

Reducing the probability of indirect LA-MRSA transmission among pig herds could be interpreted as biosecurity measures for humans visiting more than one herd on the same day. This control measure also showed a limiting effect on the between-herd spread (Tables 4–7). Humans visiting a pig herd could carry LA-MRSA for a period of a few hours up to 2 days^21^. Regulations to ensure that farm visitors (veterinarians, advisors, technicians, guests) wear masks might help to lower the risk of transmission of LA-MRSA to another farm^21^. In addition, a waiting period between visits of two pig herds might decrease the risk of pathogen transmission as well, as recommended for instance in the United States^22^.

Although pig movements might play a role in the transmission of LA-MRSA among pig herds^10,11,14,23^, movement restrictions only seemed to lead to a marginal reduction in herd prevalence. Additionally, it would be necessary to test all herds to ensure that this intervention was effective. This would require logistical and financial resources that might not be reasonable in relation to the effects predicted by the model. In addition, there is no perfect method for testing herds for LA-MRSA; in the current model, a sensitivity of 78% and a specificity of 100% were used. Despite the high specificity of the available tests^18^, the sensitivity is relatively low, leading to many false negative results. False-negative herds may jeopardise the movement restrictions and might be responsible for the low effectiveness of this strategy.

Eradication (i.e., depopulation followed by cleaning and disinfection) of LA-MRSA in herds that test positive for LA-MRSA could be an option to reduce the herd prevalence. We assumed that only small proportions of herds that tested positive for LA-MRSA would initiate an eradication programme. Depopulation and re-stocking large proportions of pig herds might lead to ethical and economic issues. In Norway, where the prevalence of LA-MRSA was low, eradication at herd level helped to limit the spread of LA-MRSA on a national level^24^. Voluntary eradication only marginally reduced the prevalence. However, eradication without the combination of movement restrictions poses the risk of re-introduction, especially when the herd prevalence is high, which might explain the low effectiveness of this control measure. In contrast, voluntary eradication combined with movement restrictions based on four tests per year led to one of the largest reductions in the predicted herd prevalence. This example highlights how intensive control measures might have had reduced the spread of LA-MRSA in Denmark. However, increasing the proportion of herds to be eradicated might be unrealistic as it would affect Danish pig production and lead to substantial economic losses^25^.

The effects of eradication on the herd prevalence are highly dependent on the proportion of herds that initiate an eradication process. Cost-benefit analyses must be included in the decision process when setting up control programmes that involve eradication efforts. In addition, the risk of re-introduction must be taken into account, for example by combining eradication with movement restrictions to minimise this risk. The role of environmental contamination in the spread of LA-MRSA has not yet been conclusively determined and therefore might influence the effectiveness of control programmes.

Herds initiating the voluntary eradication process were chosen randomly. It is therefore possible that herds registering no or only a few out-going contacts might have been selected. Prioritising herds with a high number of out-going movements or with a large out-going contact chain might increase the effects of the eradication process, as clearing these herds would prevent more herds from receiving LA-MRSA via animal movements.

For simplicity, we assumed that all herds were tested on the same day when the herd LA-MRSA status was established. In reality, herds would be tested within a certain time period (of 3 months or 1 year). This could influence the effect of movement restrictions and voluntary eradication as varying the time points at which LA-MRSA-positive herds are identified might result in control measures starting later, and transmission would still be possible until initiation of the eradication process.

We assumed that the control measures initialised in 2007 did not influence the second wave of LA-MRSA introduction in randomly chosen herds in 2009. Despite phylogenetic analysis confirming several introductions of LA-MRSA to Denmark^26^, the route of these introductions is still unknown. The effect of control measures on new introductions could therefore not be estimated. For example, LA-MRSA-positive workers could have introduced LA-MRSA in new herds, as described in Norway^24^. This route of introduction was not necessarily covered by the implemented control measures, depending on how a worker would carry the bacteria into a herd. As a result, introduction was still assumed to be possible, even under the implemented control measures.

We compared the effects of control measures starting on 1^st^ January 2007 and 1^st^ January 2010, and found that the early initialisation of control measures led to a larger reduction in the predicted herd prevalence on 31^st^ December 2012 compared to 31^st^ December 2015 (Tables 4–7). This may not be surprising, as disease control in 2007 started at a lower herd prevalence compared to initialisation in 2010 (Fig. 2). However, starting the control measures in 2010 still led to reasonably high relative reduction rates (Tables 4–7), despite an initial prevalence of 21%. The prevalence in 2016 was substantially higher at 88%^3^. Therefore, the effectiveness of these strategies from a high initial prevalence should be investigated to understand which measures or combinations could be useful in a situation with a very high prevalence, such as the current Danish situation. However, this would require a different approach to modelling animal movements, as data on registered animal movements are only available retrospectively. We therefore emphasise the importance of a region/country with a new introduction of LA-MRSA controlling/eradicating it immediately in order to prevent an endemic situation with a high prevalence.

Combinations of control measures reduced the spread of LA-MRSA, especially when all four strategies were combined. Using an extreme scenario including limiting the use of high-risk antibiotics, reducing the risk of spread via indirect contact by 75%, implementing movement restriction and culling a percentage of positive herds led to a prevalence reduction to only 6% with initiation in 2007 or 13% in 2010. This clearly shows that control of LA-MRSA can be achieved without culling all infected herds. However, it requires extreme measures, willingness from the industry and rigour in implementing these measures, otherwise high relative reduction rates might not be reached. In addition, it is important to initiate control measures as early as possible, as the effects are higher if the herd prevalence is still low.
